# Differences in parent and youth perceived neighborhood threat on nucleus accumbens-frontoparietal network resting state connectivity and alcohol sipping in children enrolled in the ABCD study

**DOI:** 10.3389/fpsyt.2023.1237163

**Published:** 2023-10-18

**Authors:** Julia C. Harris, Michael T. Liuzzi, Bo A. Malames, Christine L. Larson, Krista M. Lisdahl

**Affiliations:** Department of Psychology, University of Wisconsin-Milwaukee, Milwaukee, WI, United States

**Keywords:** resting state function connectivity, perceived threat, alcohol sipping, nucleus accumbens, frontoparietal network

## Abstract

**Purpose:**

Evidence has shown neighborhood threat (NT) as a social driver of emotional and brain development. Few studies have examined the relationship between NT and neural function. Altered functional connectivity in the nucleus accumbens (NAcc) with the frontoparietal network (FPN) has been implicated in the development of substance use, however, little is known about *perceived* NT-related brain function or downstream alcohol sipping during early adolescence. This study examined the longitudinal relationship between youth and combined youth/parent perceived NT, resting state functional connectivity (RSFC) of the NAcc-FPN, and alcohol sipping behavior during late childhood and preadolescence.

**Methods:**

This study used data (*N* = 7,744) from baseline to 2-year follow-up (FU) of the Adolescent Brain Cognitive Development Study (ABCD; Release 4.0). Relationships between youth and combined youth/parent perceive NT, alcohol sipping (baseline to two-year FU), and NAcc-FPN (left/right) connectivity, adjusting for demographics, family/peer history of alcohol use, parental monitoring and warmth, externalizing symptoms, and site, were examined in a mediation model via PROCESS in R.

**Results:**

Greater youth-reported NT at baseline was significantly associated with lower RSFC between the right (but not left) NAcc-FPN holding covariates constant (*R*^2^ = 0.01, B = −0.0019 (unstandardized), *F* (12, 7,731) = 8.649, *p* = 0.0087) and increased odds of alcohol sipping at baseline up to the two-year FU (direct effect = 0.0731, 95% CI = 0.0196, 0.1267). RSFC between the right NAcc-FPN did not significantly predict alcohol sipping at the two-year FU (b = −0.0213, SE = 0.42349, *p* = 0.9599; 95% CI = −0.8086, 0.8512). No significant relationships were observed for combined youth/parent report predicting alcohol sipping or NAcc-FPN connectivity.

**Conclusion:**

Findings suggest notable reporting differences in NT. Combined youth/parent report did not reveal significant findings; youth perceived NT was related to increased likelihood of alcohol sipping and lower neural connectivity between the right NAcc-FPN during late childhood and early adolescence. NT context – and source of reporting – may be crucial in examining links with downstream neuronal function and health behaviors. Future research should investigate reward processing and threat as the cohort ages into later adolescence.

## Introduction

### Social drivers of cognitive and brain health: perceived threat in neighborhood

According to Bronfenbrenner’s (1) ecological systems theory, child development interacts with various levels of the environment. Based on this theory, systems and socially constructed norms at all levels can impact and shape human behavior, particularly at the youth’s local environment level, including via exposure to crime, violence, and neighborhood threat. Health disparities, or major gaps in health related to factors such as race/ethnicity, socioeconomic status, or geographic location, have been identified as key health factors (2), and are critical factors in the United States to achieving health equity (3). Particularly, individuals living in more disadvantaged neighborhoods (e.g., lower perceived safety and higher levels of crime) may be disproportionately impacted by the built environment (4–7). Previous research has shown that built and natural environmental stressors, including neighborhood, school, and family threat, may be important social drivers of mental health outcomes (6, 8) and have been shown to underlie racial-ethnic disparities in health outcomes (9–12). Many posited mechanisms for racial disparities explore differences by socioeconomic status, demographic variables, and pre-existing illnesses. However, previous studies have under-emphasized the role of racial segregation, including structural and societal-level racism that create barriers to access to resources and leads to policy that disproportionately impact marginalized communities (13).

While most research has investigated socioeconomic status (SES), family environments (e.g., trauma, emotional abuse), and school environments (e.g., receiving support from teachers and peers), a growing body of research is investigating aspects of both the built and natural environment, including neighborhood aspects related to developmental trajectories and adolescent health (14–16). Characteristics of the environment, including community violence, can be a key factor in the interaction between poverty and health, environmental justice, and health equity. In a cross-sectional study, greater amounts of certain aspects of neighborhood environments, including litter and an absence of environment goods (e.g., access to a safe play for children to play), was shown to be related to increased levels of depression and anxiety (17). Increased perceptions of threat in one’s neighborhood among youth may also be a critical social driver of emotional, cognitive, and brain health and development above and beyond SES, as youth experience stressors related to their neighborhood environment (e.g., feeling unsafe, level of crime). For example, due to racial segregation, minoritized youth, including Black and Hispanic individuals, are disproportionately exposed to disadvantaged neighborhoods that can be characterized as having a heightened level of violent crime (9–11, 18). Importantly, characteristics of the neighborhood-level indicators of socioeconomic environment have been associated with risky substance use behaviors (19), which may further lead to racial and socioeconomic disparities related to the development of substance use disorders, treatment outcomes, and other health outcomes. However, many studies investigating perceptions of environment and health are limited to a cross-sectional design, thus these associations must be investigated within a longitudinal design to advance knowledge in experiences of threat related to childhood development into adolescence.

### Perceived neighborhood threat on brain functioning

Prior longitudinal research among children ages 9–10 using data from the ABCD Study has shown that elevated neighborhood disadvantage is related to worse cognitive outcomes at the two-year follow-up [e.g., memory, language ability, and vocabulary knowledge; Conley et al. (20)]. Stressful life experiences, including exposure to violence and unsafe neighborhoods, may alter reward and cognitive control networks by negatively modulating the dopaminergic reward system, which in turn can alter reward sensitivity and reward-related cognition (21). The nucleus accumbens (NAcc) is within the ventral striatum and is implicated in reward processing, sensation seeking, incentive salience, and positive reinforcement (22). Furthermore, the frontoparietal network (FPN) includes regions such as the posterior parietal cortex and the dorsolateral prefrontal cortex, regions involved in cognitive control, executive functioning, decision-making, and inhibition (23).

Related research on early life stress among children and adolescents, which may encompass witnessing violence at home or within the community, has been related to disruptions in reward processing regions, including the NAcc, ventral striatum, and prefrontal cortex (24–27). These findings have also been observed as aberrations in frontoparietal regions that underlie cognitive control, including reduced thickness and volume in the dorsolateral prefrontal cortex among children and adolescents ages 12–17 years (28, 29). Furthermore, these aberrations have been related to downstream depression and anxiety symptoms and altered reward learning among young adults [ages 18–22 years; (30–32)] and adolescents [ages 11–15 years; (33)]. Additionally, exposure to violence has been associated with reduced recruitment of the dorsal anterior cingulate cortex, a region involved in cognitive control and salience processing, in response to threat-related stimuli (34). One cross-sectional study found that among youth (ages 13–20), early child maltreatment was related to reduced functional connectivity in frontoparietal networks underlying sustained attention processes compared to individuals without history of childhood abuse (35). Early life stress and perceived threat may play an important role on the neural regions of reward sensitivity (e.g., NAcc) and regions underlying cognition (e.g., FPN). However, these studies have mainly investigated cross-sectional relationships among children and adolescents, thus a longitudinal design is necessary to explore threat associated with future risky behaviors. Given this evidence, neighborhood threat may have a unique impact on neural activity that is related to threat-related emotional processes, and, in turn, impact downstream mental health outcomes, such as early alcohol initiation.

### Perceived threat/environmental threat on alcohol sipping

A growing body of evidence has investigated the environment as it relates to alcohol sipping and initiation among youth (19, 36). Importantly, despite relatively low rates of alcohol sipping among youth [ages 9–11; 22.5% of individuals reported sipping alcohol; Lisdahl et al. (37)], young adolescent alcohol sipping is an important factor to investigate as it can be linked with downstream mental health outcomes including risky drinking and alcohol use disorder development (38, 39). It remains crucial to investigate the onset of alcohol sipping and alcohol use linked with later substance use, as few studies have investigated patterns among a large cohort of children and adolescents. As youth are undergoing a rapid period of cognitive, emotional, and brain development, understanding the environmental effects on alcohol sipping and subsequent substance use is imperative, as early alcohol use or sipping is associated with an increased likelihood for later substance use disorders (39–41). While there is evidence showing a significant relationship between perceived neighborhood threat and witnessing violence on adult alcohol use (42–46), few studies have identified the association between increased perceived neighborhood threat (e.g., community violence, neighborhood disorganization) and increased youth alcohol use (47, 48). However, studies are needed to investigate possible mechanisms of neighborhood threat on subsequent youth alcohol use, including neural mechanisms.

### NAcc-FPN on substance use

One possible mechanism underlying the links between neighborhood threat and downstream alcohol use sipping risk is neurobiology, particularly alterations in specific brain regions and networks that play a role in the reinforcing effect of alcohol use, including the NAcc, a central region implicated in alcohol-seeking (49, 50) and frontoparietal regions (51). Among adolescents aged 12–16, abnormal resting state functional connectivity of the NAcc is related to significantly less segregation of the cognitive control and reward networks (52). For example, a longitudinal study among children and early adolescents (ages 8–12) found in substance-naive youth, increased NAcc activation during a Monetary Incentive Delay task, a task related to anticipation of monetary rewards, was positively correlated with later substance use initiation and alcohol use problems before the age of 16 (53). Another study found that adolescents who had smaller left nucleus accumbens at baseline (ages 15–18) were more likely to initiate substance use at the two-year follow-up (54).

At the same time, the FPN has been shown to have altered functional activity after the onset of heavy binge drinking in later adolescents (55). Additionally, studies have found that among adults with substance use disorders, decreased resting state functional connectivity between the NAcc and fronto-cortical regions (including regions found in the FPN) is implicated in cognitive control [including dorsal anterior cingulate cortex, dorsolateral prefrontal cortex, and frontal operculum; Motzkin et al. (56)]. However, it is posited that these alterations may occur before alcohol use. While resting state functional connectivity studies of the FPN preceding alcohol use is limited, longitudinal studies of non-substance using youth has shown abnormal brain *activation* in various regions including the dorsolateral prefrontal cortex which in turn predicted alcohol use in mid-to-late adolescence (57, 58). Thus, aberrations in the FPN may lead to cognitive changes in youth and adolescents that may lead to alcohol initiation or subsequent alcohol use development. However, no studies to date have specifically examined the impact of youth perceived neighborhood threat on reward connectivity (e.g., NAcc) and FPN connectivity and subsequent alcohol sipping.

We will leverage the Adolescent Brain Cognitive Development (ABCD) Study to explore the impact of perceived neighborhood threat on NAcc and FPN network connectivity and downstream alcohol use in a large, diverse sample youth in the United States. For the present study, we are utilizing connectivity analyzes (vs. activation analyzes) to investigate whether two regions implicated in reward and alcohol sipping (i.e., NAcc, FPN) are functionally interconnected. The aims of this study were to examine (1) the longitudinal relationship between youth, combined youth and parent, and parent-only perceived neighborhood threat and resting state functional connectivity between reward (NAcc) and FPN network, (2) the links between neighborhood threat and early alcohol sipping, and (3) whether NAcc-FPN connectivity mediates this relationship. We hypothesized that after controlling for demographics, neighborhood SES, family and peer substance use risk factors (59–61), increased neighborhood threat would be related to increased odds of alcohol sipping, and that this relationship would be mediated by lower functional connectivity of the NAcc-FPN.

## Method

All protocols were approved by the University of California, San Diego, and local site institutional review boards (IRBs) and informed consent and assent were obtained from caregivers and youth.

### Participants

The study used baseline through two-year follow-up data collected from the Adolescent Brain Cognitive Development (ABCD) study, a diverse, national, prospective, longitudinal study that recruited 11,878 youth and their caregivers (*n* = 7,744; youth = 9–10 years old; see Supplementary Figure S1). Participants were excluded if youth are not fluent in English, had an MRI contraindication (e.g., ferromagnetic material in the body), history of a significant traumatic brain injury or major neurological disorder (e.g., seizures, cerebral palsy, etc.), sensory or motor impairments that prevent an individual’s ability to participate in the study, current medication of antipsychotics or mood stabilizers, gestational age less than 28 weeks or birth weight less than 1,200 grams, birth complications that resulted in hospitalization for more than one-month, uncorrected vision, or current diagnosis of schizophrenia, autism spectrum disorder (moderate, severe), intellectual disability, or alcohol/substance use disorder.

At baseline, youth and one caregiver completed one to two in-person sessions, in which they completed a battery of assessments including domains of mental and physical health (62), substance use (63), peer, family, culture, and environment (64), and MRI scans (65, 66). The current study utilized participants with complete demographic, residential history, and neuroimaging data [*n* = 8,667; (67)], thus missing data was not random. This project used ABCD 4.0 data release (2021) and was limited to using geo-coded primary address at baseline, as that is the most recently released data available.

### Measures

#### Perceived neighborhood threat

Perceived neighborhood threat at baseline (aged 9 and 10) youth report was taken from the ABCD Neighborhood Safety/Crime Survey (NSC) [for measurement properties and reliability, see (68, 69)] including one item (range = 1–5) asking youth to strongly agree to strongly disagree with the statement: “My neighborhood is safe from crime.” The Parent NSC survey contains three items: “My neighborhood is safe from crime; Violence is not a problem in my neighborhood; I feel safe walking in my neighborhood, day or night,” (range = 1–5; α = 0.87). The combined parent/youth used the sum of all youth- and parent-report items. Similarly in Conley et al. (20), perceived neighborhood threat was calculated the mean of all youth, parent, and combined youth/parent reported items. For correlations between neighborhood threat reports, see Supplementary Table S3. In short, all neighborhood threat report types were correlated with one another.

#### Individual level socioeconomic status (SES)

Individual Level SES was measured via parental education and household income. Parent education was measured by self-reported highest level of educational attainment of both parents. This variable was a categorical variable with the following categories. (1) < High School Diploma, (2) High School Diploma/General Educational Development, (3) Some College, (4) Bachelor, and (5) Post Graduate Degree. Household income was measured by asking the parent their overall household income. The item asked “What is your total combined family income for the past 12 months? This should include income (before taxes and deductions) from all sources, wages, rent from properties, social security, disability and veteran’s benefits, unemployment benefits, workman.” Responses included 1 = Less than USD 50,000; 2 = USD 50,000–100,000; 3 = USD 100,000+. Both measures were collected at baseline.

#### Neighborhood level SES

Using geo-coded primary residential address at baseline, variables from the American Community Survey, a five-year estimate between 2011 and 2015 were linked to individuals using the U.S. Census Tract which was used to calculate the area deprivation index (ADI) (70). The ADI is compiled of 17 sub-scores to capture neighborhood level deprivation; however, we selected median family income as a measurement of disadvantage as previously cited in other work (69, 71–73) including median family income.

#### Alcohol sipping

Upon starting the substance use module of the study visit, rules regarding confidentiality and privacy were reiterated to the youth and they were asked if they had “heard of” any drugs, including alcohol (63). If youth heard of alcohol, they completed the iSay Sipping Inventory (39), an 8-item measure of recent alcohol sipping that also characterized their first alcohol sipping experience. Participants reported whether they ever had a sip of alcohol, number of times had a sip of alcohol in lifetime, whether they sipped alcohol outside of a religious occasion (yes/no), total times had a sip of alcohol (outside of a religious setting), the age of first sip (outside of a religious context), and contextual factors related to their first use. For the present study, if the youth endorsed any sipping occasion outside of a religious occasion at any one of the baseline, one-year, and two-year follow-up visits, they were categorized as a “yes.”

#### Family and peer alcohol use

We calculated a dummy-coded variable representing biological parental history of AUD and other drug use disorder (DUD) taken from the Family History Assessment Module Screener, which was filled out by the youth’s participating parent or caregiver at the baseline timepoint [FHAM-S; (74)]. Responses included and coded as 0 = neither parent had a history of alcohol use problems; 1 = one parent had a history of alcohol problems; 2 = both parents had a history of alcohol use problems. For perceived peer alcohol use, youth were asked how many of their friends drink alcohol, with response options spanning from 1 = “none” to 5 = “all.”

#### Parental monitoring and warmth

Starting at baseline, a subset of acceptance items from the Child Report of Behavior Inventory [CRPBI, (75); see also (76)] was used to assess youth’s perceptions of caregiver acceptance and were used as covariates. Items are asked via youth report for the caregiver participating in the study, typically biological mothers, and a secondary caregiver chosen by the youth (e.g., father, grandparent). Youth respond on a three-point scale to items reflecting caregiver warmth and acceptance (e.g., “makes me feel better after talking over my worries;” “smiles at me very often”).

The Parental Monitoring Scale (77) evaluates the protective nature of caregiver knowledge of their child’s whereabouts, and the degree to which that intersects with shielding their youth from health-risk related behaviors. Details on the nature and sociodevelopmental theory behind this measure are described in Zucker et al. (64). Briefly, the Parental Monitoring measure has five questions that assess parents’ active efforts to keep track of their child’s whereabouts, at home and when they are not at home (e.g., who they are with; what they are doing). All items use a Likert scale ranging from never (1) to almost always (5). Parental monitoring and parental warmth were assessed using the means of both measures and both measures were administered at baseline.

#### Externalizing symptoms

The ABCD Study used a computerized Child Behavior Checklist, a well-established parent-report measure administered to the parents of youth at the baseline timepoint investigating common internalizing, externalizing, and social behaviors among children and adolescents (78). The present study used the externalizing subscale as a covariate. Externalizing symptom scores were calculated using the mean.

#### Neuroimaging procedures

Imaging protocols for the ABCD study have been outlined in Casey et al. (65). The nucleus accumbens was chosen *a priori* as a region of interest due to its association with substance use. At baseline, participants underwent resting-state fMRI acquisition for four 5-min resting-state blood oxygenation level dependent (BOLD) scans while keeping their eyes open and fixating on a crosshair. Complete neuroimaging data included scans that underwent processing and excluded for a variety of metrics, for example excessive head motion [time points with framewise displacement >0.2 mm; Power et al. (79)], failures in brain segmentation, poor behavioral performance, distortions or presence of severe artifacts, or presence of significant incidental findings (80). In accordance with Hagler et al. (80) we excluded cases with significant incidental findings, excessive motion, or other artifacts. Pair-wise correlations were examined for within functionally-defined cortical parcellations [e.g., frontoparietal network; Gordon et al. (81)] and a region of interest [NAcc; Fischl et al. (82)]. For details regarding the regions included in the FPN Gordon Network, in addition to analytic procedures, please see Gordon et al. (81). These correlation values were then Fisher Z-transformed (i.e., for left NAcc-FPN and right NAcc-FPN connectivity).

### Analytic plan

All variables were checked for multi-collinearity, skewness, and kurtosis. We employed three mediation models using PROCESS in R. The first mediation examined the relationship between baseline youth report of neighborhood threat and youth alcohol sipping onset (cumulative baseline to two-year follow-up), and whether this relationship was mediated by baseline NAcc-FPN (left and right, separately) connectivity. The second mediation examined the relationship between combined youth/parent report of neighborhood threat and youth alcohol sipping onset, and whether this relationship was mediated by baseline NAcc-FPN (left and right, separately) connectivity. The third mediation examined the relationship between parent report of neighborhood threat and youth alcohol sipping onset, and whether this relationship was mediated by baseline NAcc-FPN (left and right, separately) connectivity. The models adjusted for covariates linked with substance use risk (i.e., sex at birth, age, household income, parent education, median family income of the neighborhood, family, sibling, and peer history of alcohol use, parental monitoring, parental warmth, and externalizing symptoms; holding site as a random effect). The neighborhood threat and resting state connectivity variables are from baseline and the substance use outcome is a cumulative variables of alcohol sipping that occurred between the baseline time point through the two-year follow up.

## Results

For demographic information, see Table 1. Due to selection biases, we tested for any significant differences between our sample and the overall ABCD Study sample (see Supplementary Table S1). Additionally, testing did not reveal problems with multicollinearity; however, some variables are correlated with one another, as expected (see Supplementary Tables S2, S3). Youth perceived neighborhood threat was found to be associated with lower connectivity with right NAcc-FPN, *R*^2^ = 0.01, *B* = −0.0019 (unstandardized), *F* (12, 7,731) = 8.649, *p* = 0.0087; see Table 2. Additionally, neighborhood threat was associated with increased odds of alcohol sipping (Direct effect = 0.0731, 95% CI = 0.0196, 0.1267, *p* = 0.0074). The bootstrap confidence intervals derived from 5,000 samples indicated that the indirect effect coefficient was not significant, *b* = −0.0213, SE = 0.42349, 95% CI = −0.8086, 0.8512, which did not support the hypothesis that the relation between neighborhood threat and alcohol sipping is mediated by NAcc-FPN connectivity. No significant relationships were observed between neighborhood threat and left NAcc-FPN connectivity. No significant relationships were observed for combined youth and parent nor parent-only reporting predicting alcohol sipping or NAcc-FPN connectivity. In order to test for the specificity of our results (i.e., effect observed between NAcc and FPN), we included a post-hoc “control” analysis of resting state functional connectivity of the NAcc with a brain region not implicated in substance use (i.e., visual cortex). These results were not significant and are included in the Supplementary material.

**Table 1:** Demographic characteristics.

	Total sample (*N* = 7,744)
*Sex, n (%)*	
Female	3,818 (49.30)
Male	3,926 (50.70)
*Age, months [years], M (SD)*	119.33 [9.94] (7.57)
*Household income, n (%)*	
< $50 K	1,960 (25.30)
≥ $50 and ≤ 100 K	3,540 (45.71)
≥ $100 K	2,244 (28.99)
*Parent education, n (%)*	
< High school diploma	244 (3.15)
High school diploma/general education development	519 (6.70)
Some college	1,875 (24.21)
Bachelor’s degree	2,137 (27.60)
Post graduate degree	2,969 (38.34)
*Race, n (%)*^a^	
White	5,394 (69.65)
Black	869 (11.22)
Asian	142 (1.83)
AIAN/NHPI	46 (0.59)
Other	261 (3.37)
Multi-racial	948 (12.24)
*Hispanic, n (%)*	
Yes	1,404 (18.13)
No	6,251 (80.72)
*Alcohol sipping, n (%)*	
Yes	1,818 (23.48)
No	5,926 (76.52)
*Median family income, M (SD)*	$79,845.43 ($35,281.70)
*Youth reported neighborhood crime, M (SD)*	1.91 (1.04)
*Parent and youth reported neighborhood crime, M (SD)*	2.00 (0.80)

a Race may not include entire sample of 7,744 due to missing data (e.g., refusing to answer).

**Table 2:** Model summary of the indirect effect of perceived neighborhood threat on alcohol sipping.

Predictor	Outcome					
	*M – RSFC (right NAcc-FPN)*			*Y* – Alcohol Sipping		
	*B*	*SE B*	*p*	*B*	*SE B*	*p*
Neighborhood threat	−0.0019	0.0007	0.0087*	0.0731^	0.0273^	0.0074^*
RSFC (right NAcc-FPN)	–	–	–	0.0213^	0.4234^	0.9599^
Constant	−0.0240	0.0100	0.0164	−1.0885	0.3637	0.0028

			95% Confidence Interval	
	*B*	*SE*	Lower limit	Upper limit
Direct effect	0.0731	0.0273	0.0196	0.1267*
Indirect effect	−0.0000	0.0009	−0.0019	0.0017

*significant threshold level is *p* < 0.05.

^log odds form.

B estimates are unstandardized.

## Discussion

Previous research has shown that lower neighborhood safety and increased threat is a potential social driver of emotional health and brain development. The goal of the present study was to provide a preliminary investigation of the effect of perceived neighborhood threat, both youth and combined youth and parent report, on alcohol sipping during late childhood and preadolescence, and whether this relationship was mediated by nucleus accumbens-frontoparietal network (NAcc-FPN) resting state functional connectivity (RSFC). Our first hypothesis was supported in that this study found that greater youth-reported neighborhood threat was significantly associated with lower RSFC between the right NAcc-FPN, and increased odds of alcohol sipping at baseline and up to the two-year follow-up. However, our second hypothesis was not supported because there were no significant relationships observed for NAcc-FPN RSFC on alcohol sipping or evidence for mediation. Interestingly, there were no significant relationships found between *combined* youth and parent report or parent-only report of neighborhood threat on brain connectivity nor alcohol sipping. These findings underscore the importance of understanding how neighborhood threat can contribute to both neuronal connectivity and downstream alcohol use; however, the specific biomarker of NAcc-FPN did not mediate the relationship of threat and downstream alcohol sipping. Thus, future studies should investigate other potential neuronal markers of alcohol initiation in the context of neighborhood threat exposure.

### Perceived neighborhood threat on RSFC of NAcc-FPN

Our results support our first hypothesis and demonstrate greater perceived neighborhood threat is associated with lower connectivity between the right NAcc-FPN. This is consistent with other findings that have found greater variability in functional connectivity between the NAcc-FPN (i.e., central executive network) in a sample of cannabis users during a cue exposure task (83). Furthermore, decreased connectivity between the NAcc and frontoparietal network, including the dorsolateral prefrontal cortex, has been implicated in downstream psychopathology via emotion regulation, reward motivation, and decision-making (84–86), as well as anhedonia in patients with major depressive disorder (87). Another study found individuals with posttraumatic stress disorder displayed increased negative RSFC between areas of the FPN (dorsolateral prefrontal cortex) and the precuneus, a reward related brain region (88). Perceived neighborhood threat may be a form of chronic stress and has important implications for brain regions related to psychopathology. Furthermore, chronic stress has been found to be linked with reduced dopamine release (21, 89). The dopamine system may be a potential mechanism in the functional connectivity between FPN and NAcc which may be associated with reduced inhibitory control (90, 91).

Of note, our findings found a significant association between perceived neighborhood threat and lower *right* NAcc-FPN, but not left NAcc-FPN. While prior research has yet to explore the lateralized effects of neighborhood threat, the present findings present preliminary evidence that perceptions of neighborhood threat may uniquely impact the right NAcc. Future research can build on the present study by further investigating these effects.

### Perceived neighborhood threat on alcohol sipping

Our findings showed that perceived neighborhood threat, via youth report, was associated with increased odds of alcohol sipping. This is consistent with findings from a longitudinal study from baseline to two-year follow-up among children ages 10–13 found alcohol use initiation was significantly more common among children reporting trauma exposure and post-traumatic stress disorder symptoms (92). Early trauma and life adversity is related to problems in emotional regulation, impulsivity, decision-making, and executive function, which may lead to downstream health behaviors including using alcohol to cope with life stressors (93–95). There are several possible mechanisms underlying this relationship. Acute stress is linked with an increase in dopamine levels in the NAcc; however over time, chronic exposure to early life stress leads to blunted dopamine transmission in the NAcc, which may alter reward sensitivity (96). Thus, perceived neighborhood threat may alter reward sensitivity via downregulation of the dopamine system, which may contribute to stress sensitization (89), or a higher sensitivity to stress in which minor stressors are increasingly likely to trigger downstream mental health outcomes. Individuals with life stressors, such as neighborhood threat, and diminished reward processing may have compounded risk over time for problematic alcohol use trajectories (49). However, at this time-point neuronal signaling in these regions did not directly predict alcohol sipping, or mediate the relationship between perceived neighborhood threat and early alcohol experimentation; thus, additional research is needed to identify the neuronal markers underling this relationship. Further, given that these findings were among 9-and 12-year-olds with very minimal alcohol sipping, it’s important for future research to investigate across the developmental span to observe the impact of neuronal changes throughout adolescence.

Another potential mechanism by which neighborhood may impact adolescent health is through impacts on physical activity. Some studies have investigated positive perceptions of the neighborhood (e.g., safety, walkability, and accessibility to greenspace) as predictors for higher rates of physical activity which, in turn, may reduce depression and anxiety and increase other mental health benefits (97). Our findings are consistent with previous studies that found neighborhood factors including perceived neighborhood crime or violence associated with increased levels of alcohol use including binge drinking, hazardous alcohol use, and likelihood of alcohol use disorder among adults (98–100) and binge drinking initiation among youth ages 12–19 (101). Alcohol initiation is related to multiple aspects across social contexts, including parental substance use disorder, family dynamics, and peer relationships (39, 59, 61). Our findings extend this research as the ABCD Study to demonstrate that youth’s perceptions of neighborhood safety are also linked with early alcohol experimentation, even after controlling for known substance use risk factors.

### Youth vs. combined youth/parent reporting of neighborhood threat

Caregiver and youth reports on behavioral and mental health outcomes have discordance, particularly for perception of the environment (102). Some studies have alluded to caregivers having more insight and awareness of the environment, which may impact their monitoring and parenting styles (103). While some research has reported youth perceptions as a more robust predictor of mental health outcomes over and above county-level crime reports, findings are mixed as other studies have linked the importance of using census-level data on the neighborhood as a predictor of developmental outcomes, including mental health (18, 104, 105). Interestingly, here combined youth and parent report was not a predictor even though youth report alone was. Youth may be more accurate in reporting perceived threat in the neighborhood context as they may be increasing in autonomy as they get older and spend more time outdoors independently and within neighborhood spaces relative to their parents. Thus, it is suggested to obtain both parent and youth perception, in addition to objective reports on crime level according to census data, in order to tease apart the best predictor for mental health outcomes.

### Limitations

The present study has several limitations that should be noted. First, although this study indicates that perceived neighborhood threat may be linked with youth neuronal signaling and alcohol sipping, future studies should investigate the interaction between perceived neighborhood and other types of threat. For example, other forms of threat that should be investigated include family threat [e.g., family conflict seen through anger and aggression, neglect, and abuse; Wang and Degol (106)] and school threat [e.g., academic, interpersonal relationships between students, and institutional environment; (107)]. Importantly, family threat and school threat are associated with substance use outcomes such increased smoking, alcohol abuse, and drug abuse. Thus, the interpretation of these findings is limited to specific neighborhood threat (e.g., violence and crime within the neighborhood) as youth environments and contexts are dynamic across development and may experience varying levels of threat across settings (20). Second, the youth perceived measure of threat was limited to only one item (i.e., “My neighborhood is safe from crime.”) and is not specific to type of threat or exposure in the neighborhood. While this variable was used similarly to Conley et al. (20), there may be limitations to the construct validity of this variable. While there is only a low correlation between the neighborhood threat reports (i.e., youth and parent), we still observed expected associations between youth neighborhood threat and parent neighborhood threat with the demographic variables and other covariates. Furthermore, our findings remained consistent in that youth perceived threat predicted alcohol sipping, while combined youth and parent and parent-only perceived threat did not. This may suggest the importance of investigating longitudinal links from youth report rather than parent report on mental health outcomes and brain development. Another possible explanation could be due to the noise in youth report (i.e., limited to one question). The combined parent and youth report may have reduced measurement error due to containing two additional questions to the youth report. Future studies should employ youth self-report measures that include additional items. Third, due to the narrow scope of this study, we limited our covariates included in the Area Deprivation Index that have been found to be some of the more robust predictors of brain structure and neurocognition, including median level neighborhood income (72). However, future studies should investigate other potential confounding variables that span beyond the scope of neighborhood level income. Additionally, due to missing data and quality control of the fMRI scans, we used a sub-sample of the overall ABCD Study, there may be a certain amount of selection bias in our sample. However, this is one of the largest samples (*n* = 7,744) to investigate perceived threat on resting state functional connectivity of the NAcc-FPN. Furthermore, our study investigated a longitudinal relationship between neighborhood threat to neuronal connectivity and downstream alcohol sipping, however there is a potential for bidirectional effects including substance use on neuronal connectivity and further subsequent substance use problems. Indeed, there is evidence that binge drinking, heavy drinking or AUD diagnosis is linked with neurocognitive deficits (108); however, less is known about very low-level use and neurocognition in this preadolescent/early adolescent age range. Thus, future studies should continue to investigate this ordering of the variables in later cohorts of the ABCD Study. Finally, the present study did not investigate potential sex or racial/ethnic differences. We did not examine racial or ethnicity differences, we instead focused on social drivers of health, as they are more specific risk factors than proxy variables such as race or ethnicity. Future studies may, however, explore the intersectionality with sex at birth and gender identity. Given the potential for these differences (e.g., by sex assigned at birth, socioeconomic differences by race/ethnicity), future studies would benefit from an exploration of these variables as they related to neighborhood threat, brain connectivity, and alcohol supping in adolescents.

## Conclusion

Our results preliminarily demonstrate that perceived neighborhood threat is associated with lower connectivity between the NAcc and FPN at baseline and up to the two-year follow-up. Further, youth report of perceived neighborhood threat was linked with increased odds of alcohol sipping 2 years later. Neighborhood threat context – and source of reporting – may be crucial in examining links with downstream early adolescent neural function and health behaviors. These findings may lead to further understanding of the neural and biobehavioral mechanisms that lead to alcohol sipping and subsequent development and risk for substance use disorders. Future studies will need to further investigate task-related data in brain regions related to reward processing as the ABCD cohort ages into later adolescence. Additionally, it will be particularly useful to conduct whole-brain analyzes to investigate how the NAcc functionally interacts with the rest of the brain as it relates to alcohol sipping. Public health efforts should prioritize bolstering community-based violence interventions and protective factors to mitigate risk for substance use, for example funding initiatives that offer community engagement opportunities to youth, especially in marginalized communities disproportionately impacted by hazardous alcohol use and alcohol use disorder. Additionally, public health efforts should prioritize legislation to reduce neighborhood violence and threat.

## Data availability statement

The original contributions presented in the study are included in the article/Supplementary material, further inquiries can be directed to the corresponding author.

## Ethics statement

The studies involving humans were approved by University of California San Diego Institutional Review Board. The studies were conducted in accordance with the local legislation and institutional requirements. Written informed consent for participation in this study was provided by the participants’ legal guardians/next of kin.

## Author contributions

JH and ML: conceptualization, software, formal analysis, writing – original draft preparation. KL and CL: methodology, supervision, project administration, and funding acquisition. JH, ML, BM, KL, and CL: review and editing. All authors contributed to the article and approved the submitted version.
