# Supplementary material for: Differences in parent and youth perceived neighborhood threat on nucleus accumbens-frontoparietal network resting state connectivity and alcohol sipping in children enrolled in the ABCD study
Source: Front Psychiatry. 2023 Oct 18;14:1237163. doi: 10.3389/fpsyt.2023.1237163 (PMC10622767; doi:10.3389/fpsyt.2023.1237163)

Supplemental Material

There was no significant association found between neighborhood threat and resting state functional connectivity of the right NAcc-visual cortex, $R^2 = 0.04$, B = 0.001 (unstandardized), F(12, 7,731) = 9.5647, p = 0.6815. Additionally, neighborhood threat was associated with increased odds of alcohol sipping (Direct effect = 0.0953, 95% CI = 0.0411, 0.1495, $p = 0.0006$). The bootstrap confidence intervals derived from 5000 samples indicated that the indirect effect coefficient was not significant, b = 0.0002, SE = 0.0005, 95% CI = -0.0008, 0.0014.

Additionally, there was no significant association found between neighborhood threat and left NAcc-visual cortex, $R^2 = 0.06$, B = 0.002 (unstandardized), F(12, 7,731) = 0.1591, p = 0.6815. Additionally, neighborhood threat was associated with increased odds of alcohol sipping (Direct effect = 0.0957, 95% CI = 0.0415, 0.1499, $p = 0.0005$). The bootstrap confidence intervals derived from 5000 samples indicated that the indirect effect coefficient was not significant, b = 0.0002, SE = 0.0006, 95% CI = -0.0016, 0.0008.

Supplemental Table 1 *Comparisons of Variables between Full Sample and Study Sample*

Variable	Full Sample Information	Study Sample Information	Test Statistics
Age	*M* = 118.9798	*M* = 119.334	*t* (16,437) = 3.2117, *p* = 0.001322
Ethnicity	Hispanic = 2411<br>Non-Hispanic = 9312	Hispanic = 1404<br>Non-Hispanic = 6340	χ2 (1) = 14.506, *p* = 0.0001
Race	Asian = 275<br>Black = 1869<br>White = 7524<br>AIAN/NHPI = 78<br>Mixed = 1434<br>Other = 525	Asian = 142<br>Black = 869<br>White = 5394<br>AIAN/NHPI = 46<br>Mixed = 948<br>Other = 261	χ2 (5) = 115.037, *p* = 0.000
Youth Reported	*M* = 1.97	*M* = 1.91	

Neighborhood Crime			t (17,168) = -4.2417, p =2.23e-05
Resting State Connectivity	M = -0.0072	M = -0.0046	t (18,096) = 2.5305, p =0.0114
Median Family Income	M = $76,551.99	M = $79,845.43	t (16,914) = 6.2528, p =4.129e-10
Parental Support	M = 4.38	M = 4.41	t (17,214) = 3.6477, p =0.0002654
Parental Warmth	M = 2.735	M = 2.741	t (16,947) = 1.3897, p =0.1646
Peer Alcohol Use	M = 0.00878	M = 0.01112	t (17,811) = -1.2843, p = 0.199
Peer Full Drink	M = 0.03485	M = 0.02957	t (17,699) = -1.7039, p =0.08842
Family History of Alcohol Use	M = 0.2267	M = 0.2243	t (16,749) = -0.27919, p =0.7801
Sibling Alcohol Use	M = 0.0124	M = 0.0129	t (16,299) = 0.32726, p =0.7435
Externalizing Symptoms	M = 4.453	M = 4.055	t (17,427) = -4.8695, p =1.129e-06
Alcohol Sipping	No = 8730<br>Yes = 2495	No = 5296<br>Yes = 1818	χ2 (1) = 4.301, p = 0.0381
Sex	Female = 5680<br>Male = 6196	Female = 3818<br>Male =3296	χ2 (1) = 4.084, p = 0.0433
Household Income	< $50K = 3233 >$50 and<100k = 3071 >$100K = 4564	< $50K = 1960 >$50 and<100k = 3071<br>>$100K = 4564	χ2 (2) = 45.835, p = 0.000
Parental Education	< High School Diploma = 593<br>High School Diploma/GED = 1132<br>Some College = 3079<br>Bachelor's Degree = 3015<br>Post Graduate Degree = 4043	< High School Diploma = 244<br>High School Diploma/GED = 519<br>Some College = 1875<br>Bachelor's Degree = 2137<br>Post Graduate Degree = 2969	χ2 (4) = 120.234, p = 0.000

Supplemental Table 2 *Statistics of Collinearity*

Variable	Collinearity Statistics	
	Tolerance	Variance Inflation Factor
Age	0.982	1.019
Neighborhood Threat	0.895	1.117
Resting State Connectivity	0.984	1.016
Sex	0.960	1.042
Household Income	0.541	1.847
Education	0.593	1.688
Median Family Income	0.694	1.440
Parental Monitoring	0.809	1.237
Parental Support	0.852	1.174
Peer Alcohol Use	0.829	1.207
Peer Full Drink	0.830	1.205
Family History of Alcohol Use	0.948	1.054
Sibling Alcohol Use	0.984	1.017
Externalizing Symptoms	0.934	1.071

Supplemental Table 3 *Bivariate Correlations of all Variables*

	NT (youth)	NT (combined)	NT (parent)	Age	RSFC	Median Family Income	Parental Monitoring	Parental Support	Externalizing Symptoms
NT (youth)	-	0.570**	0.288**	-0.020	-0.057**	-0.238**	-0.157**	-0.148**	0.093**
NT (combined)		-	0.951**	-0.026*	-0.059**	-0.391**	-0.093**	-0.078**	0.152**
NT (parent)			-	-0.023*	-0.048**	-0.367**	-0.050**	-0.036*	0.142**
Age				-	0.034*	0.048**	0.107**	0.025*	-0.019
RSFC					-	0.085**	0.014	0.018	-0.030*
Median Family Income						-	0.111**	0.037**	-0.102**
Parental Monitoring							-	0.366**	-0.115**
Parental Support								-	-0.112**
Externalizing Symptoms									-

Note. ** $p < .001$, * $p < .05$

Supplemental Figure 1 *Chart Depicting Exclusion Criteria of Study Sample from Full Sample*

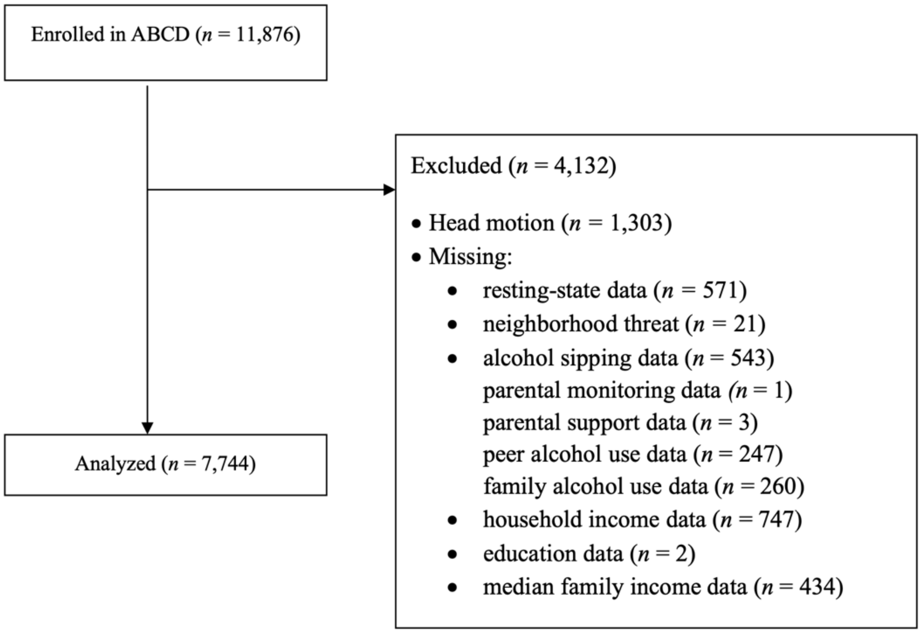

Supplement: Supplementary file 1 [file Data_Sheet_1.pdf]
